# Microwave-Assisted Synthesis of Reduced Graphene Oxide with Hollow Nanostructure for Application to Lithium-Ion Batteries

**DOI:** 10.3390/nano12091507

**Published:** 2022-04-28

**Authors:** Minseop Lee, Seung-Min Paek

**Affiliations:** Department of Chemistry, Kyungpook National University, Daegu 41566, Korea; shlee6697@naver.com

**Keywords:** reduced graphene oxide, lithium-ion batteries, Exfoliation, hollow spheres, layer-by-layer self-assembly

## Abstract

In this study, reduced graphene oxide (RGO) with a hollow nanostructure was successfully synthesized by layer-by-layer self-assembly using electrostatic interactions and van der Waals forces between building blocks, and its lithium storage characteristics were investigated. After 800 cycles at a current density of 1 A/g, the microwave-irradiated RGO hollow spheres (MRGO-HS) maintained a capacity of 626 mA h/g. In addition, when the charge/discharge capacity was measured stepwise in the current density range of 0.1–2 A/g, the discharge capacity of the RGO rapidly decreased to 156 mA h/g even at the current density of 2 A/g, whereas MRGO-HS provided a capacity of 252 mA h/g. Even after the current density was restored at a current density of 0.1 A/g, the MRGO-HS capacity was maintained to be 827 mA h/g at the 100th cycle, which is close to the original reversible capacity. Thus, MRGO-HS provides a higher capacity and better rate capability than those of traditionally synthesized RGO.

## 1. Introduction

With the development of rechargeable energy storage technology, lithium-ion storage has been widely used in various electric devices [1,2]. Therefore, electrodes with high current densities and high rate capabilities are becoming more important. Graphite, a commonly used lithium-ion battery (LIB) anode material, has a limited theoretical capacity (372 mA h/g) and poor rate capability. Moreover, because of the limitations of low energy density, meeting the social demands for various LIB applications is quite difficult [3]. Therefore, various types of graphite carbon anodes have been proposed to solve this problem.

The graphitization degree, structural disturbance, and porosity characteristics of carbon materials greatly influence the ability and mechanism of lithium-ion intercalation. On this basis, new graphite carbon anodes with disordered structures, which are advantageous for lithium-ion intercalation/deintercalation and for increasing interlayer distances, have been continuously proposed. As it has already been reported, higher lithium storage capacities can be obtained by controlling the layer structure of graphene nanosheet (GNS) materials, and the structural control of nanosheet materials can greatly affect the performance of LIBs [4]. In addition, various GNSs, such as porous films and graphene flowers, have been studied to improve the electrochemical performance by modifying structural characteristics, including the morphology of the active materials [5,6]. In this regard, hollow spheres with thin shells and large d-spacings can provide a large amount of electrochemically active sites, which eventually results in a large energy density and an enhanced rate capability [7,8,9].

**Figure 1:**
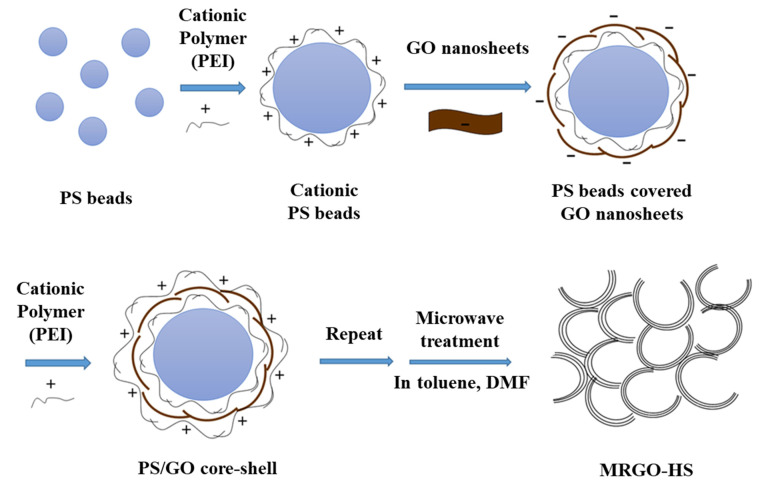
Schematic illustration of the MRGO-HS synthesis.

In this work, by using layer-by-layer self-assembly and subsequent microwave irradiation, reduced graphene oxide RGO with a hollow nanostructure was synthesized with a wider interlayer distance than that of chemically reduced graphene oxide, as seen in Figure 1. The synthesized MRGO-HS electrode and chemically reduced graphene oxide had reversible discharge capacities of 600 and 100 mA h/g, respectively, after 800 cycles when the current density is 1 A/g. That is, MRGO-HS showed more advanced lithium-ion storage performance and stability than those of conventionally synthesized RGO. In this study, we analyzed the lithium storage mechanism of MRGO-HS compared with that of chemically RGO and found that pseudocapacitive effects contribute greatly to high energy density and high rate capability. In this experiment, we developed a new synthesis method for MRGO-HS with enlarged interlayer spacings. It was analyzed how these hollow nanostructures can have excellent electrochemical performance as LIB’s anode material.

## 2. Materials and Methods

### 2.1. Synthesis of Graphene Oxide (GO)

GO was synthesized with reference to the modified Hummer’s method [10]. We added 1.0 g of graphite powder and 0.75 g of sodium nitrate into a beaker. Thereafter, we slowly added 75 mL of sulfuric acid to the beaker, and the mixture was stirred slowly while being placed in an ice bath. Next, 5 g of potassium permanganate powder was added over 1 h. We removed the mixture from the ice bath after stirring for 2 h, and it was stirred for 5 days. After reacting for 5 days, a light brown solution was obtained. Thereafter, the obtained solution was subjected to a washing process. The washing solution was separately prepared by mixing 968 mL of distilled water, 16 mL of sulfuric acid, and 16 mL of hydrogen peroxide. After the washing was finished, the solution was washed eight more times with a 1:1 distilled water and ethanol solution.

### 2.2. Synthesis of RGO

RGO as a control group was prepared using the conventional chemical reduction method using hydrazine [11]. GO (1.0 g) was dispersed in 1 L of distilled water under sonication. The temperature of the colloidal suspension of GO was kept at 90 °C, and then, 10 mL of hydrazine (hydrazine monohydrate) was added to GO suspension, and the resulting colloidal suspension was stirred for 24 h. After the reaction was completed, the suspension was cooled to room temperature and then centrifuged. The supernatant was discarded, and a solid sample was obtained. The obtained sample was washed five times with a 1:1 solution of distilled water and ethanol.

### 2.3. Synthesis of MRGO-HS

The synthesis process for MRGO-HS is shown in Figure 1. Polystyrene (PS)/GO core–shell nanostructures were synthesized using van der Waals and/or electrostatic interaction between building blocks. PS beads synthesized by previously reported methods using styrene monomers were used as templates for the core–shell structures [12]. The PS beads (0.6 g) were suspended in a mixed solution of distilled water (150 mL) and polyethyleneimine (PEI, 0.07 mL) through sonication. After this, PS beads coated with PEI obtained through centrifugation were washed five times with distilled water and then dispersed again in distilled water (150 mL). Next, 10 mL (4 g/L) of a colloidal dispersion of GO was added to the PS bead suspension positively charged by the PEI coating. The PS/GO core–shell obtained through centrifugation were negatively charged and coated with positively charged PEI again, and the above process was repeated eight times to obtain PS/GO core–shell with 8 repeated layers of GO. Thereafter, the solid product collected by centrifugation was dried at room temperature for 2 days and then subjected to microwave treatment for 10 min in a dimethylformamide solvent. Most PS beads used as templates disappeared after this process but were microwaved for an additional 5 min in a toluene solvent for a more complete reduction of GO. Finally, the obtained MRGO-HS was washed five times with acetone and then dried in an electric oven at 60 °C for 1 day.

### 2.4. Physico-Chemical Characterizations

The crystal structure of the samples was investigated by X-ray diffraction (XRD) using Bruker D2 phaser (Bruker Corp., Billerica, MA, USA) with Cu-Kα radiation (λ = 1.54056 Å) in the range of 4° to 70° (2*θ*). Microstructures, such as the morphology and the interlayer distance of MRGO-HS, were determined using a field-emission scanning electron microscope (Hitachi SU8220, HITACHI, Tokyo, Japan) and a high-resolution transmission electron microscope (HRTEM) (Titan G2 ChemiSTEM, FEI Company, Hillsboro, OR, USA) with a 200-keV acceleration voltage. We also measured the Fourier transform infrared (FTIR) (Thermo Scientific Nicolet iS5, Thermo Fisher Scientific, Waltham, MA, USA) and Raman (Renishaw inVia reflex, Wotton-under-Edge, UK) spectra using KBr pellets to analyze the carbon bonds before and after the reduction of the synthesized samples. In addition, we used X-ray photoelectron spectroscopy (XPS) (Quantera SXM, ULVAC-PHI, Yokohama, Japan) to analyze the atomic composition of the surface. N_2_ adsorption-desorption experiments were performed using Autosorb-iQ and Quadrasorb SI instruments (Quantachrome, Boynton Beach, FA, USA), and pre-experiment samples were dehydrated at 100 °C for 12 h using a vacuum oven. Thermogravimetric analysis (TGA) experiments in an ambient condition were conducted using an STA-S1000 analyzer (SCINCO, Co., Ltd, Seoul, Korea).

### 2.5. Electrochemical Performance Measurement and Electrochemical Kinetics Analysis

Charge and discharge experiments to investigate lithium-ion storage characteristics were performed using battery test equipment (Maccor K4300, Tulsa, OK, USA). MRGO-HS, which was used as an active material, was made in the form of a slurry for casting to copper foil. N-methyl-2-pyrrolidone was used for mixing the active material, conductive carbon, and polyvinylidene fluoride in a ratio of 7:2:1. The resulting slurry was cast flat throughout the copper foil. Then, it was dried for 12 h in a vacuum oven at 100 °C. The coin-type half-cell (type 2032) was manufactured in a glove box filled with Ar gas. The battery cell consisted of an active electrode, a 3501-type separator, and a lithium metal counter electrode. The internal electrolyte used 1M LiPF_6_. The charge/discharge tests of the assembled cell were conducted at various current densities using a Maccor K4300 instrument. In addition, the electrochemical property was analyzed through a cyclic voltammetry (CV) test using WMPG1000 (WonA Tech, Seoul, Korea), and sweep speeds of 0.1, 0.3, 0.5, and 0.7 mV/s were performed in the voltage window of 0.01–3.0 V. The electrical conductivity and lithium-ion diffusion behavior of the anode materials were analyzed using electrochemical impedance spectroscopy (EIS; ZiVE SP2: WonA Tech, Seoul, Korea) data. The electrochemical experiments, such as electrochemical performance, CV, and EIS, were analyzed at room temperature.

## 3. Results

### 3.1. Structural Analysis

**Figure 2:**
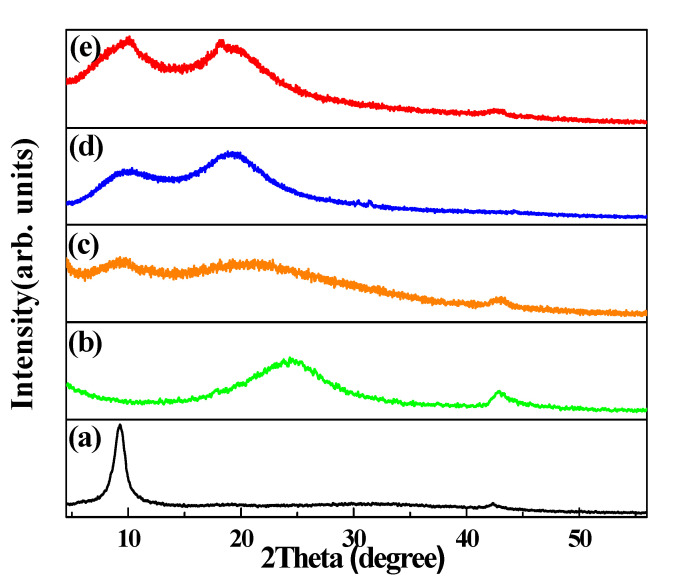
XRD patterns of (**a**) graphene oxide (GO), (**b**) reduced GO (RGO), (**c**) MRGO-HS, (**d**) PS beads, and (**e**) PS/GO core–shell.

In Figure 2, the sharp diffraction peaks appearing at 9.4° in the XRD of GO correspond to reflections from the (002) planes [13,14,15]. The formation of oxygen functional groups on the surface of the graphene layer can increase the interlayer spacing, with an extended d-spacing (0.95 nm) compared with that in raw graphite (0.34 nm) [16,17,18]. The weak peak at 44° can be indexed to the turbostratic structure, typically found in randomly reassembled 2-dimensional materials. For the RGO samples, the broad reflection peak from (002) planes appears around 24.6°, corresponding to a d-spacing of 0.37 nm, because the alignment of the sheets along the crystallographic *c*-direction is not uniform [19]. Moreover, the absence of a peak at 9.4° indicates that the oxygen functional groups underwent chemical reduction and were removed from the RGO samples [20,21,22,23]. MRGO-HS has a wide (002) peak centered at 2*θ* = 23°. The average basal spacing of MRGO-HS was turned out to be 0.39 nm, which is much larger than that of RGO (0.37 nm). It is expected that the enlarged interlayer spacing of MRGO-HS could be quite advantageous for the fast transport of lithium ions, resulting in enhanced electrochemical lithium storage properties.

**Figure 3:**
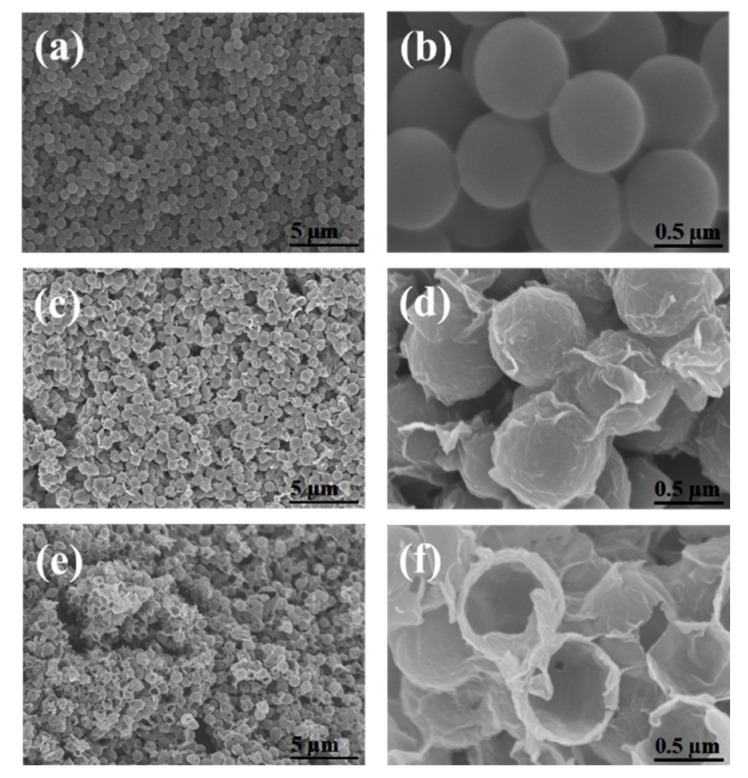
SEM images of the (**a**,**b**) PS beads, (**c**,**d**) PS/GO core–shell, and (**e**,**f**) MRGO-HS.

Figure 3 shows the SEM images of the (a,b) PS beads, (c,d) PS/GO core–shell, and (e,f) MRGO-HS. Figure 2a,b shows that the PS beads with smooth surface have an average diameter of 0.7 μm. The SEM image of the PS/GO core–shell in Figure 2c,d shows that GO was uniformly coated on the smooth surface of the PS beads to form a rough surface. After removing the PS beads by microwave treatment, the spherical structures of MRGO-HS were maintained, as shown in Figure 3e,f, and we confirmed that the shell of the hollow sphere was composed of several stacked nanosheets. MRGO-HS also had the form of interconnected hollow microspheres with rough surfaces.

**Figure 4:**
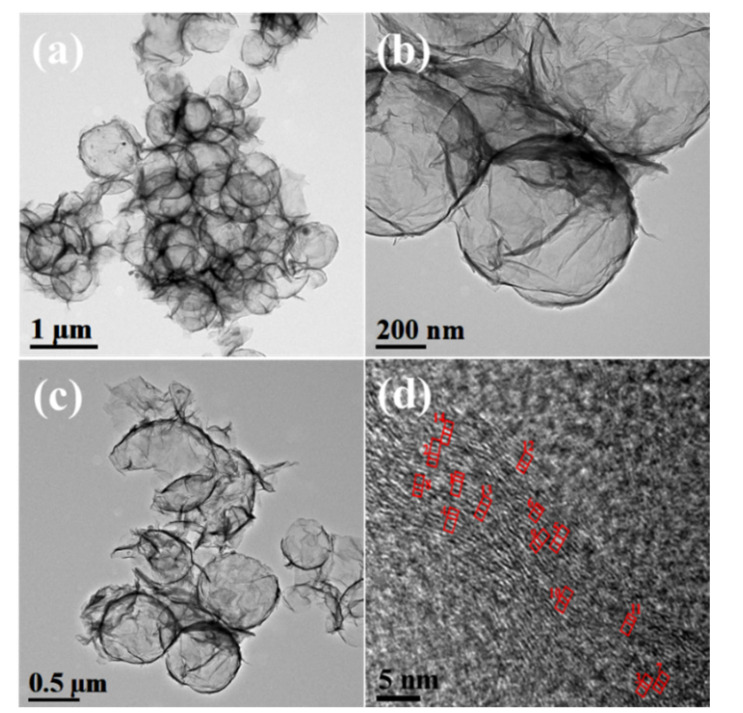
TEM images of (**a**–**c**) MRGO-HS. (**d**) HRTEM image of MRGO-HS with the multi-layer structure indicated by the red line.

**Figure 5:**
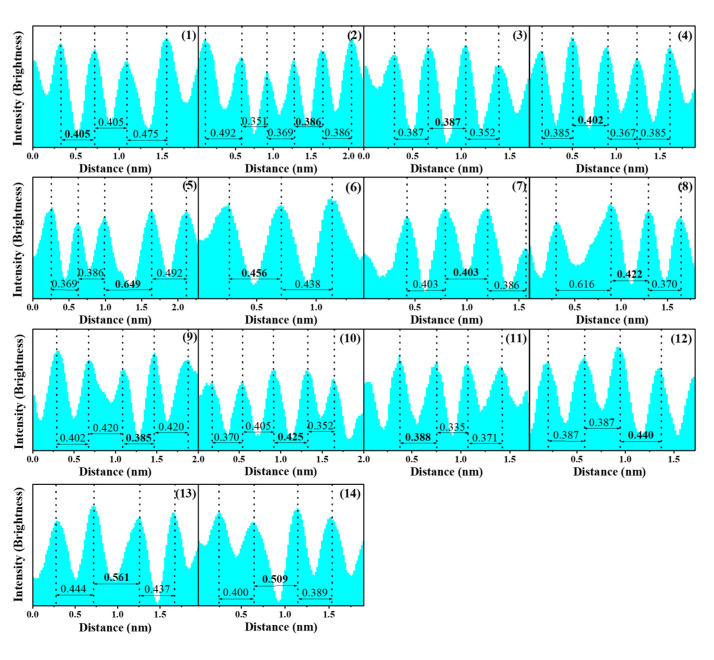
Brightness profile of the red lines in Figure 4d.

The morphological features of MRGO-HS are also shown in the HRTEM images. Figure 4 shows the HRTEM images of MRGO-HS at low and high magnification. The thickness of the shell at the relatively rough outer surface is estimated to be about 20 nm. In addition, the average diameter of MRGO-HS is about 750 nm. The HRTEM image and brightness profile of MRGO-HS in Figure 5 show that the average interlayer distance of the observed part is 0.43 nm, which is much larger than that obtained from XRD analysis. The (002) peak appearing in the XRD of MRGO-HS is very weak and broad. The measured d-value from the (002) XRD peak can be close to the approximate average value, but the interlayer spacing from the local HRTEM measurement is inevitably different from the average d-value. However, this result represents a larger basal spacing of MRGO-HS than those in conventional graphene-based nanomaterials [4]. Such a wide basal spacing of the graphene layers is expected to provide both effective lithium-ion transport and additional insertion sites.

**Figure 6:**
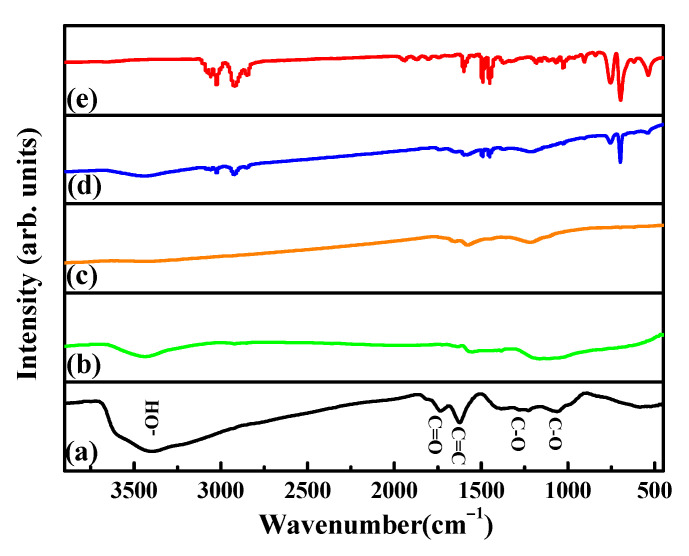
FTIR spectra of (**a**) GO, (**b**) RGO, (**c**) MRGO-HS, (**d**) PS beads, and (**e**) PS/GO core–shell.

Figure 6 shows the FTIR spectra of (a) GO, (b) RGO, (c) MRGO-HS, (d) PS beads, and (e) PS/GO core–shell. The FTIR peaks at 3405, 1064, 1228, 1390, 1630, and 1735 cm^−1^ are shown in the case of the GO spectrum, which imply the existence of C–O, C–O–C, C–OH, C = C, and C = O (carboxylic acid and carbonyl moieties). After the reduction process, the FTIR peak that appeared in GO almost disappeared or decreased greatly [24,25,26]. In the spectrum of Figure 6b, the peak at 1575 cm^−1^ represents C = C and is displayed because of the unoxidized graphene domain. The absorption peak at 1174 cm^−1^ may correspond to the remaining O–H and C–O stretching. These FTIR data show that the oxygen functional groups present in the GO were removed after the reduction process, which is in good agreement with the XPS data to be discussed in XPS analysis [27,28,29]. In the spectrum of MRGO-HS, characteristic peaks at 3030, 2930, 1490, 1450, 761, and 701 cm^−1^ appearing in the FTIR spectrum of the (d) PS beads and (e) PS/GO core–shell are not observed. This indicates that the PS beads used as template materials can be effectively removed by microwave treatment with an organic solvent. Moreover, when compared with the GO spectrum, all functional groups of MRGO-HS almost disappeared. Therefore, not only the PS beads used as templates were effectively removed using microwave irradiation in an organic solvent but also the oxygen functional groups in GO.

**Figure 7:**
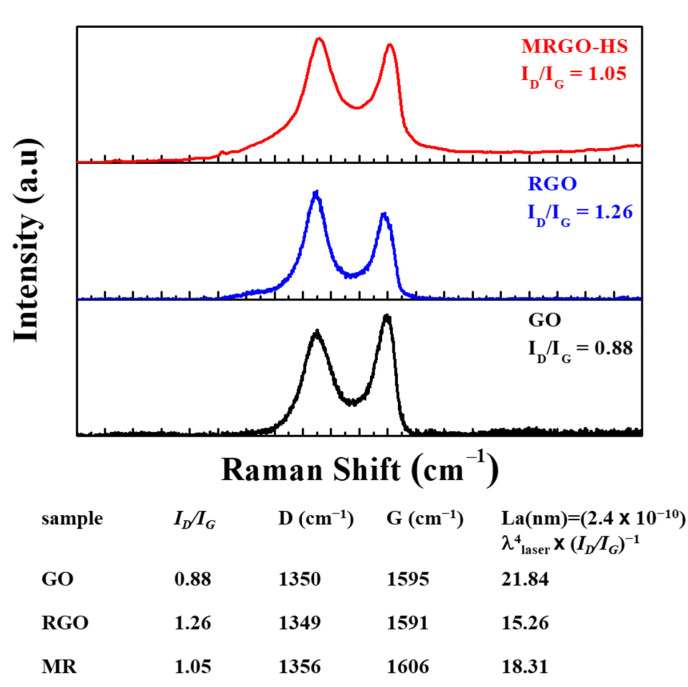
The Raman spectra of GO, RGO, and MRGO-HS.

Structural changes in the process of reducing GO can be seen in the Raman spectrum (Figure 7). As already reported, the Raman spectrum of graphite can show the only prominent G-band peak at 1581 cm^−1^ [30]. The G-band is associated with the E_2_g vibrational mode of the sp^2^ carbon atom with a hexagonal structure. In the GO spectrum, a broad G-band can be seen at 1595 cm^−1^, and a D-band representing a failure due to structural defects can be seen at 1350 cm^−1^. This leads to smaller sp^2^ domains during oxidation. The D-band is related to the disordered A_1g_ breathing mode of the aromatic ring [31,32,33,34]. The MRGO-HS Raman spectra show G- and D-bands at 1606 and 1356 cm^−1^, respectively. MRGO-HS (1.05) also shows a higher *I*_D_/*I*_G_ ratio than that of GO (0.88). Comparing the average sizes of the sp^2^ domains using Knight’s empirical equation [35,36,37], the size of sp^2^ domains in MRGO-HS have smaller than those in GO. Although the size after the microwave reduction process was small, more new graphitic domains were created than those present in GO [38]. Therefore, the RGO obtained by the reduction treatment shows that the C = C portion of the graphitic structure has been restored, and the graphitization degree has increased. RGO also observed a similar change in the *I*_D_/*I*_G_ ratio, which was higher (1.26) than that of MRGO-HS. Therefore, larger sp^2^ domains could be restored from GO when using microwave reduction rather than chemical reduction [39,40,41,42].

**Figure 8:**
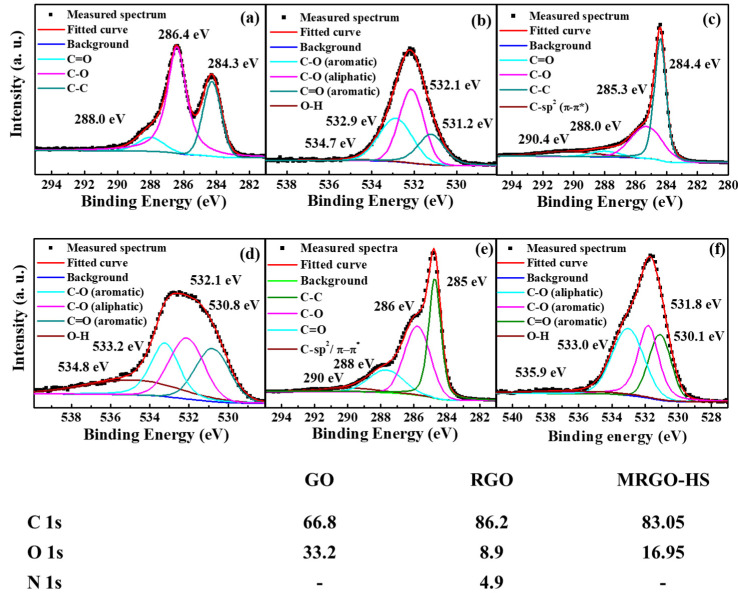
C 1s and O 1s core level X-ray photoemission spectra of the (**a**,**b**) GO, (**c**,**d**) RGO, and (**e**,**f**) MRGO-HS samples. The table below the spectra shows the atomic percentages of C, O, and N in GO, RGO, and MRGO-HS in the XPS analysis.

The XPS analysis was used to examine the chemical composition before and after the reduction of the synthesized samples. The XPSPEAK41 software was used for the deconvolution of the C 1s and O 1s peaks. S-peak-type and Shirley-type backgrounds were used. In addition, parameters such as the full width at half-maximum, peak position, and area that can be adjusted during deconvolution were used, and joints using Lorentzian–Gaussian functions were performed. Comparing the atomic percentage of each sample, the ratio of carbon is further increased in RGO and MRGO-HS compared with that in GO, indicating a higher carbon-to-oxygen (C/O) ratio. Figure 8 shows the XPS spectrum of C 1s for each sample. In GO’s C 1s XPS spectrum, we can see three trough peaks at 284 eV from non-oxygen ring (C–C) carbons because of the graphite sp^2^ framework. In addition, a peak due to epoxy and alkoxy groups is observed at 286 eV, and carbonyl and carboxyl functional groups can be confirmed through the peak at 289 eV. Therefore, it can be known that the oxygen-containing functional groups in GO were bonded to the surface of the graphene layer, confirming the successful synthesis of GO [43,44,45]. After the reduction process, peaks from oxygen-containing functional groups can also be observed in the C 1s spectra of RGO and MRGO-HS. However, it can be seen that the peak intensity for oxygen-containing functional groups decreased more rapidly than that of GO, and a peak showing a much sharper contribution of (C–C) bond than that of GO at 285 eV can be seen. Therefore, these XPS results are similar to the epoxy and alkoxy functional groups and carbonyl groups from the GO surface after going through chemical and microwave-assisted reduction processes. We can confirm that the sp^2^ C–C bond of the graphite structure was almost restored by showing that the oxygen functional group was removed at a considerable rate [46,47]. In the case of RGO and MRGO-HS, a π–π* shake-up satellite peak widely displayed at 290 eV is observed. This characterizes the aromatic and delocalized π conjugation in graphite and confirms the successful reduction process [48,49,50].

The oxygen-containing functional groups of each sample were further investigated using the O 1s spectrum in Figure 8. Four peaks by oxygen functional groups can be observed around 531, 532, 533, and 535 eV of the separated spectra, respectively. These are due to single bonds in aromatic carbon and oxygen in quinones, single bonds in carbon and oxygen, carbonyl, and carboxyl groups (C=O), carboxyl groups, and internal moisture (O–H) [51,52,53].

Therefore, the above XPS analysis results show that microwave-assisted reduction can be effectively used to restore the graphite framework from GO.

**Figure 9:**
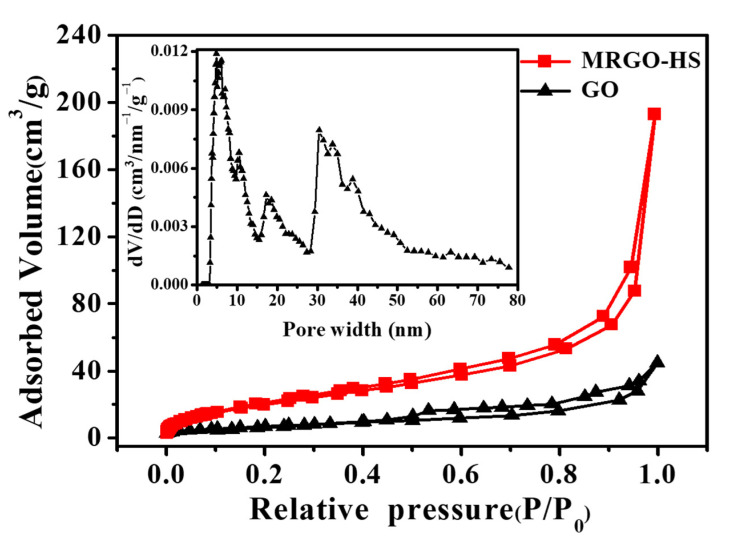
N_2_ adsorption/desorption isotherms of GO and MRGO-HS. The inset image is the pore size distribution curves of MRGO-HS.

The porosity and surface area of MRGO-HS were studied using N_2_ adsorption–desorption isotherm measurements. MRGO-HS showed isotherms characteristic of Brunauer-Deming-Deming-Teller-IV-shaped mesoporous materials with H_3_-type hysteresis loops. This is usually associated with capillary condensation [54]. In MRGO-HS in Figure 9, N_2_ adsorption in low relative pressure (P/P_0_ < 0.6) is rarely seen, and the adsorption and desorption curves are consistent as adsorption and desorption consist of single-layer adsorption and a fairly reversible process. Conversely, in the relatively high-pressure region (P/P0 > 0.6), it shows a fairly steep adsorption point, and an H3-type hysteresis loop appears. These types of isotherm and hysteresis are mainly manifested by parallel walls or open slits and narrow neck capillaries. Therefore, it can be seen that most of the pores of MRGO-HS are slit-shaped pores slit by the aggregation of nanosheets. In contrast, MRGO-HS showed only weak nitrogen adsorption up to 1-bar relative pressure and no pronounced hysteresis. This is due to the multiple layers of GO filled with oxygen functional groups. The isotherm of GO was classified as BDDT type III, which is characteristic of nonporous materials. The Brunauer–Emmett–Teller specific surface area was 20 m^2^/g in the case of GO and 80 m^2^/g for MRGO-HS, confirming the increase in the surface area caused by the microwave-assisted reduction process. In addition, for MRGO-HS compared with GO, the N_2_ isotherm shows an increased slope of P/P_0_ between 0.05 and 0.5, indicating the presence of developed mesopores. The pore size distribution of MRGO-HS was calculated using the Barrett–Joyner–Halenda (BJH) method. As shown in the inset of Figure 9, MRGO-HS formed mesopores developed by the stacks of hollow hemispheres [55,56].

**Figure 10:**
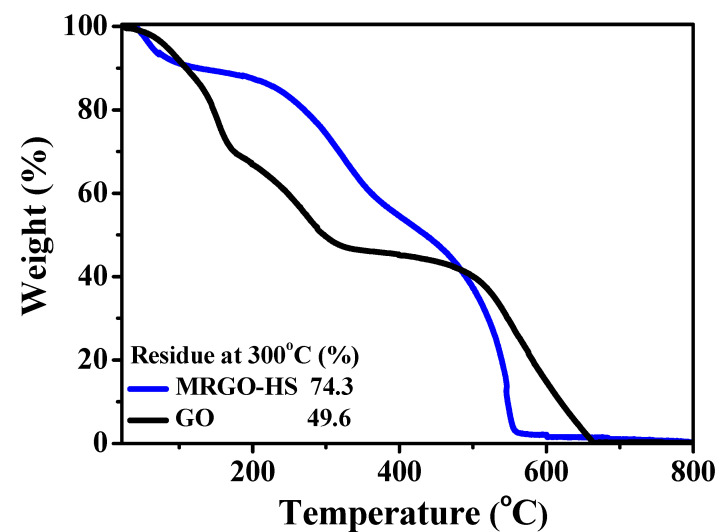
TGA curves of MRGO-HS and GO.

Figure 10 shows the TG curves for MRGO-HS and GO. MRGO-HS has a weight loss of about 24.7%, smaller than that for GO at 300 °C. This indicates that the amount of oxygen-containing functional groups was effectively removed by the microwave treatment, which greatly improved the thermal stability of MRGO-HS [57]. Therefore, it can be seen that the PS/GO core–shell nanostructures were sufficiently reduced by the microwave treatment. MRGO-HS shows four stages of weight loss. The evaporation of moisture within MRGO-HS and GO up to 100 °C initially yielded a mass loss of about 12%. Thereafter, a second mass loss section appears up to 300 °C due to the pyrolysis of the residual oxygen-containing functional groups that remained after the microwave treatment. Gradual weight loss can then be seen below 350 °C. This is due to the combustion of some of the remaining PS beads and the pyrolysis of residual oxygen-containing functional groups, particularly carboxyl groups. The graphene shell of MRGO-HS began to pyrolyze at 400 °C and was completely burned around 600 °C [30].

### 3.2. Electrochemical Performance of RGO and MRGO-HS

**Figure 11:**
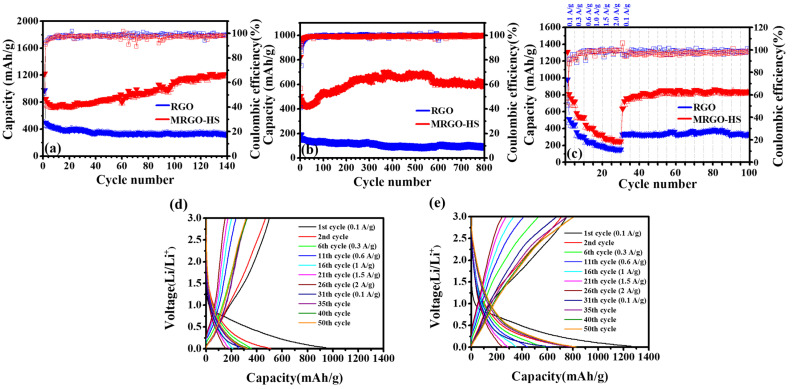
(**a**) Cycling performance and coulombic efficiency of RGO and MRGO-HS (at the current density of 0.1 A/g), (**b**) at the current density of 1 A/g, (**c**) at various current densities. Galvanostatic charge/discharge curves of (**d**) RGO, and (**e**) MRGO-HS between 0.01 and 3.0 V at various current densities.

Figure 11 shows the results of the charge/discharge tests of MRGO-HS and RGO. The prolonged cycling performance of MRGO-HS can be shown by maintaining a discharge-specific capacity of 600 mA h/g at a fairly high current density of 1 A/g after 800 cycles. It is also stable but shows a fairly high capacity increase. The initial Coulomb efficiencies of RGO and MRGO-HS were 41% and 57%, respectively. The formation of the solid electrolyte interphase (SEI) seen in the first cycle caused a loss of capacity and, consequently, a low initial irreversible capacity [42]. After 10 cycles, the CE of MRGO-HS was maintained at 97% or more. Even after 800 cycles, a CE of 99% or more is exhibited, and the very stable cycling of MRGO-HS can be confirmed. These increased capacities and high CE are due to the broader interlayer spacing caused by the microwave treatment and the structural properties of the hollow spheres that can buffer the volume change of the electrode material while forming a stable SEI and increasing the access of the electrolyte. Figure 11 shows the rate capability of MRGO-HS and RGO at different current densities. MRGO-HS showed a high initial discharge capacity of 1310 mA h/g. After the 30th cycle, the current density returned to 0.1 A/g, and the discharge capacity of MRGO-HS remained at 800 mA h/g. Therefore, we can see that the speed performance of MRGO-HS was greatly improved.

**Figure 12:**
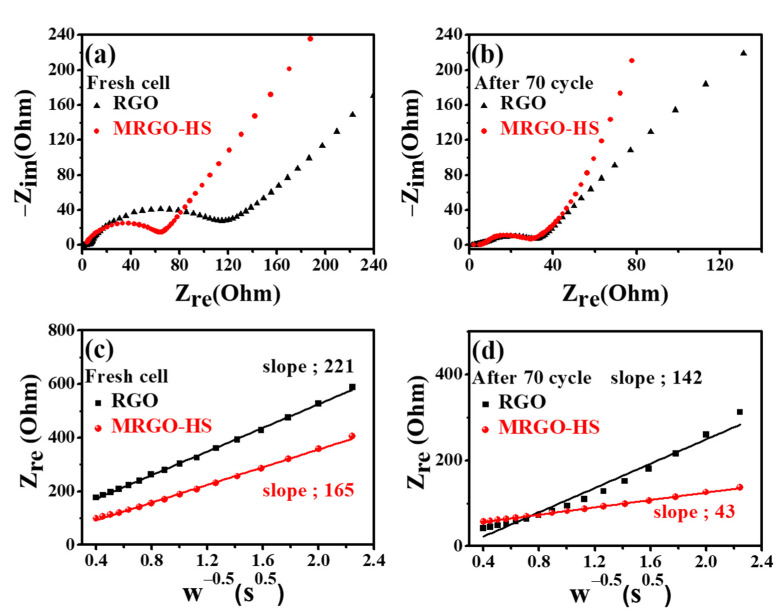
(**a**) Nyquist plots for a fresh cell and (**b**) Nyquist plots after 70 cycles. Warburg plots for a fresh cell (**c**) and after 70 cycles (**d**).

EIS data were measured to confirm the improved conductivity of MRGO-HS. Figure 12 shows a flat semicircle shape in the high-frequency region and an inclined line appearing in the low-frequency region shown in the graph of each sample. In the Nyquist plots of a fresh cell that has not undergone a charge/discharge, the semicircle diameter of MRGO-HS was much smaller than that of RGO. Thus, *R*_ct_ (charge transfer resistance) was much smaller for the MRGO-HS electrode. Further details are discussed in a previous study [58]. After 70 cycles, the *R*_ct_ values of the two electrodes decreased significantly. Because of the surface activation, *R*_ct_ was greatly reduced, and no slick growth of the SEI layer was observed on the two electrodes up to 70 cycles [59]. Because MRGO-HS has a thin-shell structure, it ensures improved accessibility of the electrolyte and has less aggregation between graphene sheets than RGO and a wider gap between layers. Therefore, MRGO-HS has good electron transport channels even in the initial cycle. On the other hand, in the fresh cell, RGO has a higher R_ct_ value compared to MRGO-HS. However, after 70 cycles, R_ct_ value of RGO is similar to that of MRGO-HS. This is because both materials consist of similar graphene frameworks. Therefore, when the electron transport channel of the graphene framework is activated by the reversible insertion/deinsertion reaction of lithium ions in RGO, the charge transfer behavior can be increased, similar to that of MRGO-HS. Figure 12c,d is a Warburg plot showing the relationship between the impedance (*Z*_re_) in the low-frequency region and ω^−0.5^. This relationship is described by Equation (1).

*Z*_re_ = *R*_ct_ + *R*_s_ + σω^−^^0.5^ (1)

The line displayed in the low-frequency region is due to the diffusion of lithium ions, also called Warburg diffusion. The smaller the slope of the Warburg plot determined via least-squares fitting, the faster the diffusion of the lithium cations. As shown in Figure 12c,d, the lithium-ion diffusion rate of MRGO-HS is higher than that of RGO, implying a higher lithium-ion conductivity for the MRGO-HS electrode [59]. The increase in discharge capacity of MRGO-HS is due to the improved accessibility of lithium ions to the graphene framework of MRGO-HS during cycling. In Figure 12c,d, after 70 cycles, the Warburg plots of the MRGO-HS electrode decreased significantly compared to the fresh cell, confirming that the ionic conductivity was significantly improved. Therefore, it can be seen that the ionic transport of lithium ions can increase upon successive cycling. As expected, the MRGO-HS electrode had faster ion diffusion because of the advantages of the hollow sphere structures and the increased interlayer spacing. Consequently, better electrical conductivity and a faster charge transfer process than those of RGO could be achieved.

### 3.3. Electrochemical Kinetics and Pseudocapacitive Property Characterization of MRGO-HS

**Figure 13:**
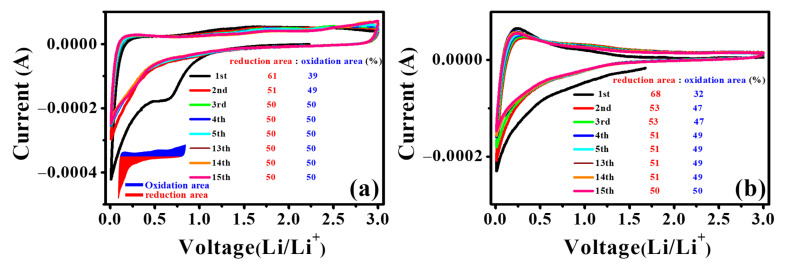
Area covered under the CV curves of (**a**) MRGO-HS and (**b**) RGO.

Figure 13 shows the region of the circulating voltage–current curve and the initial 15 cycle oxidation and reduction cycle curves. The displayed area reflects the Li^+^ and electrons involved in the electrochemical reaction. In Figure 13, the area (61%) of the first discharge curve of MRGO-HS is larger than the area (31%) in the first oxidation process. This is due to the formation of SEI and is reflected in the initial high discharge capacity. After the second cycle, the area of the reduction cycle and the area of the oxidation cycle each converged to 50%. This means that the lithium ions involved mostly returned during the oxidation process when the first discharge occurred. This is also reflected in the high Coulomb efficiency and reliable capacity maintenance shown by the charge/discharge data. The ratio of the area of the first discharge curve compared with the area during the oxidation process of RGO was higher than that of MRGO-HS. This meant more SEI formation. Moreover, during the second and third cycles, the area (53%) of the reduction process was higher than that of the oxidation process (47%). This means that the lithium ions used during the reduction cycle did not completely return during the oxidation process [60,61]. In the RGO oxidation and reduction cycle curves, the area ratio after 14 cycles converged to 50%. This confirms that MRGO-HS showed improved cycling stability compared with that of RGO from the charge/discharge experimental data. Moreover, the wider reduction and oxidation cycle regions of MRGO-HS than those of RGO indicate that more lithium ions are involved in the reaction, implying a greater charge capacity. 

**Figure 14:**
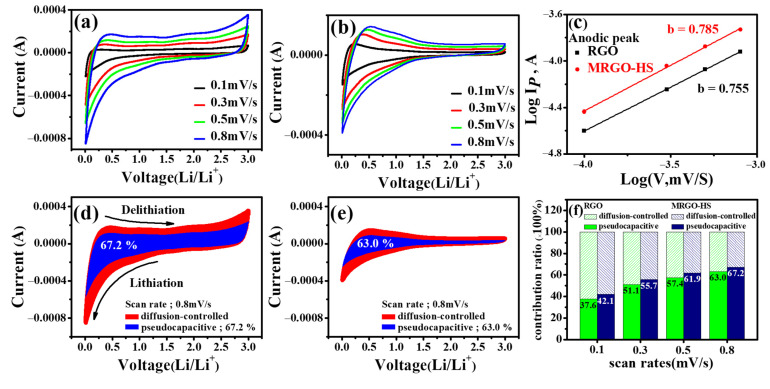
CV curves of (**a**) MRGO-HS and (**b**) RGO at different scan rates. (**c**) The log(*i*) versus log(*v*) plots of MRGO-HS and RGO. Pseudocapacitive (blue area) and diffusion (red area) contribution to the charge storage of (**d**) MRGO-HS and (**e**) RGO at 0.8 mV/s. (**f**) Proportion of pseudocapacitive contribution at different scan rates of MRGO-HS and RGO.

CV measurements were made at various sweep speeds of 0.1, 0.3, 0.5, and 0.8 mV/s, thereby comparing the dynamics of MRGO-HS and RGO to further investigate their capacitive behaviors. The CV curve at each sweep rate showed a similar shape (Figure 14a,b). The relationship between the current (*i*) to be measured and the scan speed (*ν*) is expressed by Equations (2) and (3).

I = *a*ν^*b*^ (2)

log(*i*) = *b*log(*ν*) + log(*a*) (3)

In the given equations, *a* and *b* are empirical parameters. As the *b* value is displayed closer to 0.5, the capacity contribution of the diffusion control process is more dominant. In addition, the closer the value of *b* is to 1, the greater the surface-controlled current, indicating an ideal capacitive behavior [62]. Figure 14c is an MRGO-HS log(*ν*) (mV/s) versus log(*i*) (mA/g) plot measured at other sweep speeds from 0.1 to 0.8 mV/s. The value of *b* of the peak at the anode can be obtained from the slope. The *b* value appears at 0.785, a value greater than 0.5, which means that the storage and release of Li^+^ are governed by pseudocapacitive and diffusion-controlled Faraday processes [63]. The *b* value of RGO was calculated as 0.755. Therefore, the advantage of the hollow sphere structures in pseudocapacitive process can be seen by comparing the slopes in Figure 14c. As described above, the total contribution can be divided into the pseudocapacitive process and the diffusion-controlled process. Therefore, investigating what governs electrochemical reactions is necessary to compare the electrode performance and determine the mechanism of the charging/discharging process. In the case of Equations (2) and (3), the total contribution can be divided into the two parts (a pseudocapacitive effect part and a diffusion-controlled part) as in Equation (4).

*I* = *k*_1_*ν* + *k*_2_*ν*^0.5^ (4)

In the case of *k*_1_*ν* in Equation (4), the ratio of the current due to the pseudocapacitive effect is shown, and *k*_2_*ν*^0.5^ shows the current response by a diffusion-controlled process. *k*_1_ and *k*_2_ are constant values. Therefore, *k* can be obtained through the relationship between *i* (current in the CV curve) and *ν* (scanning speed), as shown in the following equation [63,64,65,66].

*i*/*ν*^0.5^ = *k*_1_*ν*^0.5^ + *k*_2_ (5)

Figure 14d–f depicts the contribution of capacitance determined by calculating the value of *k* at scan speeds of 0.1, 0.3, 0.5, and 0.8 mV/s. We compared the ratio of the MRGO-HS and RGO current due to the pseudocapacitive effect. The contribution ratio of pseudocapacitance increased with increasing scan speed, and the contribution ratios of the pseudocapacitance in MRGO-HS were higher than those in RGO at all measured scan speeds [67]. These electrochemical properties are the advantages of the nested hollow microsphere structures of MRGO-HS. Such structures improved the contact between the electrolyte and the electrode surface, provided more active sites for storing lithium ions, and shortened the diffusion path for ionic transport [64]. Therefore, we confirmed that MRGO-HS showed better rate performance than that of RGO because of its relatively high contribution of pseudocapacitive processes.

## 4. Conclusions

We proposed a process for producing MRGO-HS using layer-by-layer self-assembly and subsequent microwave irradiation in organic solvents. As expected, the structure of the hollow spheres showed stable cycling and speed performance compared with those of the conventional RGO and showed improved charge transfer efficiency. In addition, MRGO-HS showed improved pseudocapacitance behavior and clearly demonstrated its structural advantages. Moreover, the superior speed performance associated with the pseudocapacitive effect can be expected in the anodes of next-generation LIBs. Therefore, MRGO-HS with an extended interlayer gap compared with that of existing graphene-based materials can be evaluated as a very good structure for the anode materials of LIBs.

## Data Availability

The data presented in this study are available on request from the corresponding author.
